# PRAME predicting outcome in uveal melanoma

**DOI:** 10.1002/2056-4538.70116

**Published:** 2026-10-14

**Authors:** Sally Owens, Naomi Walsh, Noel Horgan, Patrick Murtagh, Katie Hanratty, Grainne Finegan, Laura Ivers, John Crown, Susan Kennedy

**Affiliations:** ^1^ Life Sciences Institute, School of Biotechnology Dublin City University Dublin Ireland; ^2^ National Ophthalmic Pathology Laboratory, Department of Pathology Dublin Ireland; ^3^ Ocular Oncology Service, Department of Ophthalmology Research Foundation Royal Victoria Eye & Ear Hospital Dublin Ireland; ^4^ Department of Medical Oncology, St. Vincent's University Hospital Dublin Ireland

**Keywords:** uveal melanoma, PRAME, BAP1, metastatic risk, immunohistochemistry

## Abstract

Uveal melanoma (UM) is the most common primary intra‐ocular malignancy in adults and carries a high risk of metastatic disease, affecting approximately 50% of patients despite advances in local tumour control. PRAME (PReferentially expressed Antigen in MElanoma) has emerged as a marker for metastatic risk; however, evidence is largely derived from mRNA expression studies rather than routine immunohistochemistry (IHC). This study evaluates PRAME protein expression by IHC in a large UM cohort and examines its association with clinicopathological features and survival outcomes. A total of 403 UM cases were included. PRAME IHC was performed on tissue microarrays (TMAs) and/or whole tumour sections and scored as positive based on nuclear staining in >10% of tumour cells. Associations were assessed using chi‐square and Mann–Whitney *U* tests. Survival outcomes were analysed using Kaplan–Meier curves and Cox proportional hazards models. PRAME positivity was identified in 5% of TMA cases and 40% of whole‐section cases. On TMAs, PRAME expression was associated with worse overall survival (median 26 versus 50 months; *p* = 0.03) but not metastasis‐free survival (MFS). In contrast, whole‐section PRAME expression was strongly associated with shorter MFS (median 5 versus 20 months; *p* < 0.001) and worse overall survival (median 13 versus 52 months; *p* = 0.04). In multivariate analysis including age, American Joint Committee on Cancer stage and BAP1 status, whole‐section PRAME positivity remained an independent adverse predictor of MFS (hazard ratio = 10.5, *p* = 0.002). PRAME IHC is a clinically meaningful prognostic biomarker in UM, particularly when assessed on whole tumour sections, where it provides strong independent prognostic information.

## Introduction

Uveal melanoma (UM) is the rarest form of melanoma accounting for 3–5% of cases worldwide [1]. It is the most common primary intra‐ocular malignancy in adults with an incidence rate of 5–7 individuals per million per year [2]. Rates vary by geography with the highest observed in northern Europe and the lowest in Asia. Ireland has one of the highest reported incidences worldwide at 10.2 per million per year, following Denmark, which has the highest at 11 per million per year [3]. There have been considerable advancements in the management of primary UM as radiation‐based procedures have replaced whole eye enucleations for smaller non‐eye‐blinding tumours [4]. The biggest challenge surrounding UM remains its likelihood of hematogenous metastasis, which is observed in 50% of patients within 10 years of their initial diagnosis [5, 6].

Metastatic UM (mUM) unfortunately does not respond to the same targeted therapies that are effective in patients with cutaneous or conjunctival melanoma, and efforts to date have been unsuccessful due to the immunosuppressive environment or the low tumour mutational burden seen with mUM [7]. In recent years, immunotherapy for mUM has expanded to include immune checkpoint inhibitors (ICIs) and T‐cell receptor (TCR)‐based therapies, such as the FDA‐approved tebentafusp [8]. Tebentafusp, a bispecific TCR agent targeting the gp100 antigen in HLA‐A*02:01‐positive patients, has shown improved survival and a favourable safety profile compared to ICIs. Overall, tebentafusp offers better tumour specificity and tolerability, while combination ICI therapies provide broader immune activation but at the cost of increased toxicity [8]. For HLA‐A*02:01‐negative patients, the need for alternative targeted therapies remains.

Prognostic factors in UM have been extensively studied to better predict the likelihood of progression and to stratify patients into low‐ and high‐risk groups. Clinicopathological systems, such as the American Joint Committee on Cancer (AJCC) [9], emphasise tumour diameter, thickness, and ciliary body involvement as important indicators of metastatic risk. The Cancer Genome Atlas (TCGA) [10] shows that loss of chromosome 3 and gain of chromosome 8q are strongly associated with poor outcome, while other alterations such as gain of chromosome 6p may carry protective effects. In addition, mutations in genes including *GNAQ* and *GNA11* as initiating events, and *BAP1*, *EIF1AX*, and *SF3B1* as driving mutations have refined tumour classification and prognostication [11, 12]. Given the poor outcomes associated with metastatic disease and the lack of improvements with UM specific survival, the identification of additional robust biomarkers of disease progression is essential.

A 15‐gene expression profile (GEP) established by Harbour *et al* separated UM cases into two classes – Class 1 for low risk and Class 2 for high‐risk cases [13]. Two studies by the Collaborative Ocular Oncology Group demonstrated the potential of molecular markers outperforming traditional clinical parameters in predicting metastatic risk [13, 14]. In particular, the studies investigated the importance of PRAME (PReferentially expressed Antigen in MElanoma) RNA expression as a biomarker for metastatic risk in UM, particularly in Class 1 melanomas that lack the markers of metastatic disease displayed in Class 2 tumours [14].

PRAME is a tumour‐associated antigen that was established as an indicator of poor prognosis in many cancers [15]. PRAME promotes tumour progression by binding to the retinoic acid receptor (RAR), interfering with retinoic acid signalling, which normally induces cell differentiation, growth arrest, and apoptosis [16]. As PRAME has little to no expression in healthy tissue with the exception of testis, ovary, placenta, adrenals, and endometrium, there is an interest in investigating the role of PRAME as a candidate for targeted therapies [15]. Immunohistochemistry (IHC) is an accessible way to determine the frequency of PRAME expression in melanomas that could be employed as a standard laboratory test and has been shown to be just as effective as mRNA expression studies [17].

In this study, we determine the frequency of PRAME expression in 403 primary UM tumours and compare the findings to known disease progression and overall survival (OS) outcomes. We hypothesise that detection of PRAME by IHC is as effective in predicting prognostic events as mRNA studies. As IHC is more accessible as a routine test, it would be a more practical option for histology laboratories. We also compare PRAME IHC results with BAP1 expression and monosomy 3 to assess the power of these in combination with PRAME.

## Materials and methods

### Study design

Patients diagnosed with UM in the Royal Victoria Eye and Ear Hospital (RVEEH) were recorded by the National Ophthalmic Pathology Laboratory (NOPL). Clinical, molecular and histopathological data were obtained through patient charts and information systems. Long‐term follow‐up data was obtained from the National Death Registry. Exclusion criteria were set and included insufficient clinical data or tissue. A total of 403 patients were included in the study who were diagnosed between 1965 and 2025.

Clinicopathological features assessed were patient age, sex, AJCC TNM staging (T1–T4) and cell type. Fluorescence *in situ* hybridisation (FISH) analyses provided chromosome 3 and chromosome 8 aberration status which in turn was used to sort cases into the TCGA (A‐D) classification system [10]. PRAME and BAP1 IHC staining was performed by accredited laboratories on formalin‐fixed paraffin‐embedded (FFPE) tumour whole sections and tissue microarrays (TMAs). This study was approved by the Ethics Committees of the RVEEH and Dublin City University (DCUREC/2025/175). Patients gave informed consent and permission was granted for the use of historic anonymised patient samples. The study was conducted in accordance with the Declaration of Helsinki [18].

### Immunohistochemical staining and scoring

TMAs were constructed using the Manual Tissue Arrayer (Beecher Instruments, Sun Prairie, WI, USA). Triplicate 600 μm cores from each tumour were placed across the TMA alongside non‐tumour reference samples (tonsil or liver). The minimum requirement for scoring samples was one assessable core. Whole tissues were sectioned at 4 μm thickness.

IHC for BAP1 and PRAME was carried out on the Ventana Benchmark Ultra automated platform (Ventana Medical Systems, Tucson, AZ, USA) using an alkaline phosphatase red detection system. All procedures were carried out in an ISO 15189‐accredited diagnostic laboratory. Sections were deparaffinized and underwent heat‐induced epitope retrieval using CC1 (64 min at 100 °C). Slides were incubated with a primary BAP1 antibody (sc‐28383, Santa Cruz Biotechnology, Dallas, TX, USA) at a dilution of 1:50, or with a primary antibody against PRAME (clone EPR20330; Biocare Medical, Pacheco, CA, USA; ref. AVI3252G) for 32 min at 37 °C. Signal amplification was applied, and nuclei were counterstained with Harris haematoxylin (8 min; GCC Diagnostics, UK; catalogue no. S0500) followed by a blueing reagent (Ventana Medical Systems).

PRAME and BAP1 TMA and whole‐section slides were scored by two observers in the NOPL, who were unaware of patient outcomes at the time of scoring (SK and SO). PRAME was marked as positive if nuclear staining was observed in >10% of tumour cells [17]. Specimens were marked as nuclear BAP1 positive when staining was observed in >50% of the tumour cells [19]. Both stains were then recorded on a binary system of positive or negative. Discrepancies were discussed and resolved by consensus. See supplementary material, Figure S1.

### Cytogenic analysis

FISH was performed on 100 nuclei using the Vysis CEP 3 (D3Z1, satellite III) SpectrumOrange Probe (catalogue no. 06J36‐013; Abbott Molecular Inc., Des Plaines, IL, USA) targeting the centromeric region of chromosome 3, and separately with the ZytoLight SPEC MYC/CEN 8 Dual Colour Probe (Z‐2092; ZytoVision GmbH, Bremerhaven, Germany) detecting the centromere of chromosome 8 and the MYC locus (8q24.21). The procedures followed previously published protocols [20]. All analyses were carried out in a clinically accredited laboratory. Scale bars were calibrated using the average diameter of tumour cell nuclei (approximately 7–8 μm) as an internal reference, see supplementary material, Figure S2.

### Statistical analysis

Statistical analysis was conducted in IBM SPSS v30 (IBM Corp., Armonk, NY, USA). Age was a continuous variable which was dichotomised by the median. All categorical data was analysed as binary variables. Comparisons of clinical and histopathological features of the different risk groups were performed using Pearson chi‐square and categorical variables and Mann–Whitney *U* for continuous variables. A *p* value of <0.05 was considered statistically significant.

OS was calculated from the date of operation to the last date of follow‐up or death from disease or from other causes, if known. Metastasis‐free survival (MFS) was also calculated from the date of operation to the date of metastatic disease where applicable. Date and cause of death information was reported by primary care physicians, the National Death Registry or patient charts.

The Cox proportional‐hazards model was first applied in univariate analyses to assess the association of each variable with OS and MFS. Variables that reached statistical significance in univariate analysis (*p* < 0.05) were then included as covariates in the multivariate model.

Survival curves for OS and MFS were generated using the Kaplan–Meier survival estimator, with the log‐rank test used to determine statistical significance.

## Results

### Clinical, histological, and cytogenic characteristics

A total of 403 patients from the NOPL database met the criteria for inclusion in this study. The patient clinical features are described in Table 1. There were 223 (55.3%) males and 180 (44.7%) females with a median age of 63 (48.9% were younger than the median age). Initial treatment was surgery (primary enucleation) in 367 (91%) of cases, while 36 (9%) received surgery (enucleation) post radiation therapy.

**Table 1:** Clinical and histological features of the National Ophthalmic Pathology Laboratory (NOPL) cohort

Clinical feature	*n* = 403	%
Sex		
Male	223	55.3
Female	180	44.4
Age (years)		
Median	63	
<63	197	
>63	206	
Cell type		
Spindle	152	37.7
Epithelioid	78	19.4
Mixed	135	33.5
TNM stage		
pT1	67	16.6
pT2	81	20.1
pT3	138	34.2
pT4	70	17.4
Metastasis		
Yes	179	44.4
No	224	55.6
Chromosome 3		
Monosomy	107	26.6
Disomy	132	32.8
Chromosome 8q		
Gain	135	33.5
Disomy	98	24.3
TCGA stage		
D3/D8	73	18.1
D3/8qG	54	13.4
M3/8qG	45	11.2
M3/8qG multiple	34	8.4
M3/D8	22	5.5
Outcome		
NED	172	42.7
AWD	37	9.2
DOD	143	35.5
DOC	51	12.7
OS months		
Median [range]	49	[1–587]
PFS months		
Median [range]	19	[0–124]

For all tumours, histological metrics were available. According to AJCC staging, 67 (16.6%) of tumours were classed as pT1, 81 (20.1%) as pT2, 138 (34.2%) were pT3, and 70 (17.4%) as pT4. Histologically, 152 (37.7%) of tumours were spindle cell type, 78 (19.4%) epithelioid, and 135 (33.5%) were mixed. FISH analysis was available for 239/403 (59%) patients. 107 (44.8%) of tumours exhibited monosomy of chromosome 3 (M3), and 135 (57.9%) showed gain of chromosome 8q (8qG). Similarly, 73 (30.5%) were classed as group A according to TCGA classifications, 54 (22.6%) as B, 45 (18.8%) in C, and 34 (14.2%) in group D. The TCGA classes do not include a category of M3/D8; 22 (9%) patients were in this category.

The median time to metastatic spread was 19 months (range 0–124). Of these, 179 (44.4%) patients progressed, of which 143 (35.5%) died of disease (DOD), with 37 (9.2%) currently alive with disease (AWD). Currently, 172 (42.7%) patients have no evidence of disease (NED) after treatment and 51 (12.7%) died of other causes (DOC). The median time from metastatic disease until disease‐related death was 12 months (range 0–72).

### Association between PRAME expression and clinicopathological features

PRAME IHC scores were available for all patients, 383 from TMAs and 83 from whole sections with 57 overlapping (results from both). PRAME was expressed (positive) in 19 (5%) of TMA cases and 33 (39.8%) of whole sections. The association of PRAME expression (TMA and whole section) with the relevant clinical features is displayed in Table 2. A significant association was observed between spindle cell type (*p* = 0.013) and loss of nBAP1 expression (*p* = 0.038) in the TMA cases and chromosome 8q gain (*p* = 0.009), OS (*p* = 0.009), and MFS (*p* = 0.001) in whole‐section slides.

**Table 2:** Clinical and histological features in association with IHC PRAME expression results in TMA and FFPE whole section slides

Clinical feature	TMA (*n* = 383)			Full section (*n* = 83)		
	PRAME+ (*n* = 19)	PRAME− (*n* = 364)	*p*	PRAME+ (*n* = 19)	PRAME− (*n* = 364)	*p*
Sex						
Male	11	159	0.893	17	25	0.893
Female	8	205		16	25	
Age (years)						
<63	12	183	0.273	11	23	0.251
>63	7	181		22	27	
Cell type						
Spindle	4	141	**0.013**	9	20	0.440
Epithelioid	9	65		9	13	
Mixed	6	124		12	13	
TNM stage						
pT1	1	65	0.389	3	3	0.574
pT2	4	70		8	14	
pT3	10	124		9	20	
pT4	4	59		12	13	
Chromosome 3						
Monosomy	5	92	0.420	15	25	0.772
Disomy	10	117		12	23	
Chromosome 8q						
Gain	10	113	0.407	24	21	**0.009**
Disomy	4	90		2	17	
OS months						
Median [range]	26 [12–83]	50 [1–587]	0.122	13 [4–94]	52 [7–108]	**0.009**
PFS months						
Median [range]	14 [5–39]	20 [0–124]	0.775	5 [0–15]	24 [5–69]	**0.001**
nBAP1 expression						
Expressed	5	176	**0.038**	12	27	0.173
Not expressed	14	171		17	20	

Values reaching statistical significance (*p* < 0.05) are highlighted in bold. FFPE, formalin‐fixed paraffin‐embedded; nBAP1, nuclear BAP1; OS, overall survival; PFS, progression‐free survival; TMA, tissue microarray.

### Prognostic role of PRAME expression – TMA Results

Positive PRAME expression was associated with significantly worse OS [*p* = 0.03, log rank; median OS for negative PRAME expression 50.0 months (95% CI 41.33–58.67) versus positive PRAME expression 26.0 months (95% CI 20.98–31.02)], with early separation of survival curves within the first 2 years (Figure 1A). The estimated 5‐year OS rate was 42% in PRAME‐negative tumours compared to 25% in PRAME‐positive tumours, with long‐term survival predominantly observed in PRAME‐negative cases.

**Figure 1:**
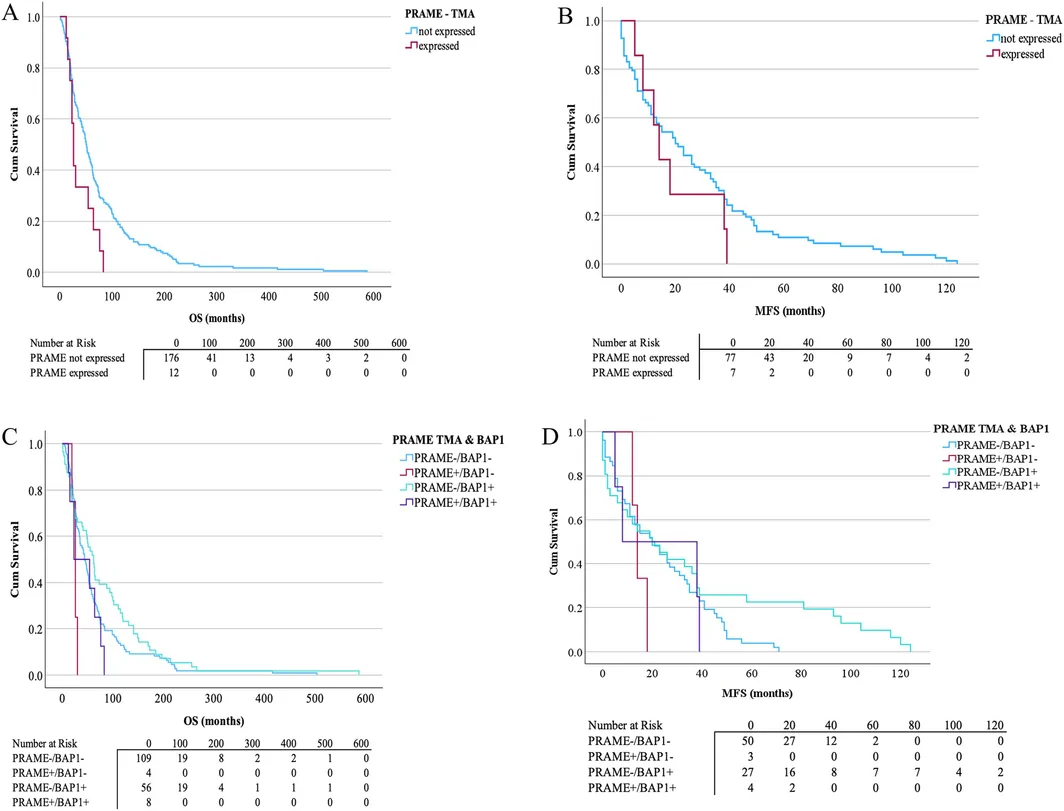
Kaplan–Meier (A) overall survival (OS) and (B) metastasis‐free survival (MFS) curves stratified by PRAME positive expression (red) and negative expression (blue). Kaplan–Meier (C) overall survival (OS) and (D) metastasis‐free survival (MFS) curves stratified by PRAME IHC results combined with BAP1 IHC results. PRAME+/BAP1− (pink), PRAME+/BAP1+ (purple), PRAME−/BAP1− (blue) and PRAME−/BAP1+ (cyan). Time is portrayed on the *x*‐axis in months. Log‐rank tests were used to compare survival between the two groups.

Although PRAME‐positive tumours showed earlier disease progression, the difference in MFS did not reach statistical significance [*p* = 0.376, log rank; median MFS for negative PRAME expression 20.0 months (95% CI 11.97–28.04) versus positive PRAME expression 14.0 months (95% CI 8.87–19.13); Figure 1B]. The 5‐year MFS rate was approximately 11% in the PRAME‐negative cohort, while the PRAME‐positive cohort reached 0% at the same interval. While PRAME expression follows a trend for worse MFS, interpretation of survival estimates is limited by the small number of PRAME‐expressing cases in the TMA cohort.

### Prognostic role of PRAME expression in combination with BAP1

BAP1 IHC was available for 379 tumours, 186 (49.1%) were nuclear BAP1 (nBAP1) negative. Of these nBAP1 negative tumours, 31 (8%) also showed PRAME expression (PRAME+/BAP1−). Seventeen (4%) were PRAME positive while also retaining nBAP1 expression (PRAME+/BAP1+).

Of the four combined groups (PRAME±/ BAP1±), the PRAME+/BAP1− group showed the worst OS and MFS, followed by the PRAME+/BAP1+ group. PRAME−/BAP1+ showed the most favourable outcomes. This observation was significant in OS [*p* = 0.036, log rank; median OS for PRAME−/BAP1− 44.0 months (95% CI 34.7–53.3), PRAME+/BAP1− 26.0 months (95% CI 20.06–31.94), PRAME−/BAP1+ 62.0 months (95% CI 48.39–75.61), PRAME+/BAP1+ 23.0 months (95% CI 0.00–59.03)] but not in MFS [median MFS for PRAME−/BAP1− 20.0 months (95% CI 9.90–30.10), PRAME+/BAP1− 14.0 months (95% CI 10.80–17.20), PRAME−/BAP1+ 20.0 months (95% CI 4.73–35.27), PRAME+/BAP1+ 8.0 months (95% CI 0.00–40.34)] (Figure 1C,D).

### PRAME IHC whole section

A subset of cases (*n* = 83) had PRAME IHC performed on the full FFPE tumour sections. Survival analyses show positive PRAME expression had a distinct worse prognosis in both OS [log rank *p* = 0.039; median OS for negative PRAME expression 52.0 months (95% CI 38.74–65.26) versus positive PRAME expression 13.0 months (95% CI 0.00–33.45)] and MFS [*p* < 0.001, log rank; median MFS for negative PRAME expression 20.0 months (95% CI 6.93–33.07) versus positive PRAME expression 5.0 months (95% CI 3.40–22.60)] (Figure 2A,B). Kaplan–Meier curves show an early separation for MFS as PRAME positive tumours metastasise. When combined with nBAP1 expression, the same trend is seen as the TMA survival curves but with significance in both OS [*p* = 0.021, log rank; median OS for PRAME−/BAP1− 52.0 months (95% CI 38.74–65.26), PRAME+/BAP1− 16.0 months (95% CI 0.00–37.47), PRAME+/BAP1+ 5.0 months (95% CI 0.00–13.82)] and MFS [*p* < 0.001, log rank; median MFS for PRAME−/BAP1− 29.0 months (95% CI 5.17–52.83), PRAME+/BAP1− 6.0 months (95% CI 1.20–10.80), PRAME−/BAP1+ 12.0 months (95% CI not estimable), PRAME+/BAP1+ (data not estimable)] (Figure 2C,D).

**Figure 2:**
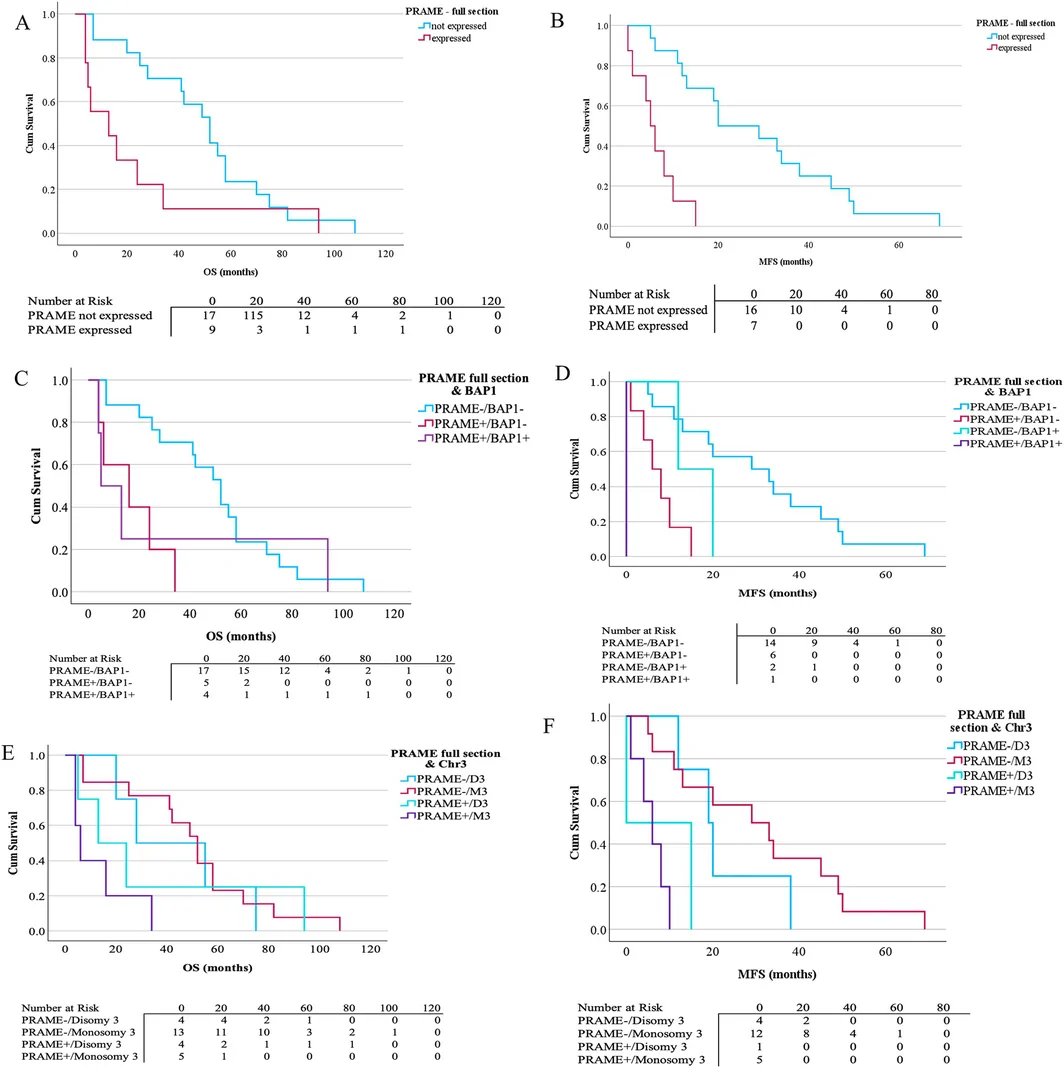
Kaplan–Meier (A) overall survival (OS) and (B) metastasis‐free survival (MFS) curves stratified by PRAME positive expression (red) and negative expression (blue) for full section FFPE slides. Kaplan–Meier (C) overall survival (OS) and (D) metastasis‐free survival (MFS) curves stratified by PRAME full section IHC results combined with BAP1 IHC results. PRAME+/BAP1− (pink), PRAME+/BAP1+ (purple), PRAME−/BAP1− (blue) and PRAME−/BAP1+ (cyan). Note in OS there is no PRAME−/BAP1+ group as no patient met the criteria for this test. Kaplan–Meier (E) overall survival (OS) and (F) metastasis‐free survival (MFS) curves stratified by PRAME full section IHC results combined with chromosome 3 status. PRAME−/Disomy 3 (blue), PRAME−/monosomy 3 (red), PRAME+/Disomy 3 (cyan) and PRAME+/monosomy 3 (purple). Time is portrayed on the *x*‐axis in months. Log‐rank tests were used to compare survival between the groups.

Given the strong association between monosomy 3 and poor outcome in UM, PRAME expression was analysed in combination with chromosome 3 status. Kaplan–Meier survival analysis showed that PRAME‐positive tumours had significantly worse outcomes for both OS [*p* = 0.008, log rank; median OS for PRAME−/D3 28.0 months (95% CI 0.00–62.30), PRAME−/M3 52.0 months (95% CI 40.54–63.46), PRAME+/D3 13.0 months (95% CI 0.00–31.62), PRAME+/M3 6.0 months (95% CI 1.71–10.29)] and MFS [*p* < 0.001, log rank; median MFS for PRAME−/D3 19.0 months (95% CI 11.16–26.84), PRAME−/M3 29.0 months (95% CI 6.93–51.07), PRAME+/D3 not estimable, PRAME+/M3 6.0 months (95% CI 1.71–10.29)] (Figure 2E,F). The PRAME+/monosomy 3 subgroup exhibited the poorest prognosis, with no survivors after 40 months. PRAME−/disomy 3 tumours showed the most favourable outcome, maintaining the highest survival and longest MFS over time. The MFS curves showed an early separation, with PRAME‐positive tumours in both chromosome 3 subgroups developing metastasis within 2 years.

When assessed in the TMA cohort, a similar trend was observed. PRAME+/monosomy 3 tumours demonstrated the worst prognosis followed by PRAME+/disomy 3 tumours, although these differences did not reach statistical significance [OS log rank *p* = 0.292, median OS for PRAME−/D3 48.0 months (95% CI 36.65–59.35), PRAME−/M3 34.0 months (95% CI 21.31–46.69), PRAME+/D3 19.0 months (95% CI 10.41–27.59), PRAME+/M3 26.0 months (not estimable); MFS log rank *p* = 0.751; median for MFS PRAME−/D3 19.0 months (95% CI 9.61–28.39), PRAME−/M3 19.0 months (95% CI 8.82–29.18), PRAME+/D3 12.0 months (95% CI 0.80–23.20), PRAME+/M3 14.0 months (95% CI 4.20–23.80)] (supplementary material, Figure S4).

In a univariate Cox proportional hazards analysis, positive PRAME expression on whole‐section IHC was strongly associated with shorter MFS (hazard ratio [HR] = 8.5, *p* < 0.001). PRAME expression assessed on TMA was not significantly associated with MFS (supplementary material, Table S1). For OS, whole‐section PRAME expression was similarly associated with worse outcome in univariate analysis (HR = 2.37, *p* = 0.047), whereas PRAME expression detected by TMA had no significant association (supplementary material, Table S1). Variables that were significant in the univariate analysis were subsequently included in multivariate Cox regression models. PRAME positivity on whole‐section IHC remained an independent predictor of shorter MFS (HR = 10.5, *p* = 0.002). In the OS multivariate model, advanced tumour stage (AJCC pT4) remained a predictor of worse OS (HR = 8.4, *p* = 0.032) (supplementary material, Table S1).

In univariate Cox regression analysis, combined PRAME/BAP1 status was significantly associated with OS in both the TMA cohort (*p* = 0.038) and the full‐section cohort (*p* = 0.022). PRAME‐positive tumours with BAP1 loss demonstrated the highest risk of death in both cohorts (TMA: HR = 3.51, *p* = 0.018; Full section: HR = 4.45, *p* = 0.11) (supplementary material, Table S2). Cox regression analysis for MFS did not reach statistical significance in the TMA cohort and could not be reliably estimated in the full‐section cohort due to low event numbers in these combination groups.

## Discussion

UM is a rare, yet deadly disease with a very high likelihood for hematogenous metastasis [21]. Patients with metastatic UM have a very low survival rate of less than 1 year [21]. The aggressive nature of metastatic disease makes risk categorisation vitally important for both surveillance and treatment management. Patients can be stratified into high and low risk groups based on clinical, histological and genetic features. GEP has emerged as a prognostication tool in UM, notably through a 15‐gene assay developed by Harbour and colleagues [14], which stratifies tumours into Class 1 (low metastatic risk) and Class 2 (high metastatic risk) categories. Class 2 tumours are strongly associated with established high‐risk features, including *BAP1* mutations and monosomy 3. To further refine risk stratification within Class 1 tumours, there is a subdivision into Class 1A for low risk and 1B for intermediate risk. This is based on differential expression of *CDH1*, a tumour suppressor gene [22] and *RAB31*, which drives drug resistance as it interferes with extracellular vesicle formation [23].

While this approach has shown prognostic value, a small percentage of tumours classified as low risk by GEP still progress. This has driven efforts to identify additional markers capable of identifying higher risk tumours within favourable classes. Field *et al* demonstrated that PRAME mRNA expression is the most significant predictor of metastatic risk among Class 1 tumours [16]. Their study showed that PRAME negative Class 1 tumours exhibited a 0% 5‐year rate of metastasis, whereas PRAME positive Class 1 tumours had a 38% rate of metastasis. This PRAME positive group was classed as intermediate risk, with a median MFS of 88 months, which is longer compared to the MFS of 32 months in Class 2 patients.

PRAME expression also correlated with chromosomal instability, including copy number gains of 1q, 6p, and 8q, supporting its relevance as a marker of tumour progression. The inclusion of PRAME allowed patients to be stratified into Class 1 PRAME− (low risk), Class 1 PRAME+ (intermediate risk), and Class 2 (high risk) [16]. Although PRAME is well established as a prognostic biomarker, it also plays a functional role in tumour progression. Recent studies indicate that PRAME may contribute to genomic instability through disruption of cohesion complex function resulting in impaired DNA damage repair and increased aneuploidy [24]. PRAME has dual transcriptional effects as both an activator and a repressor [16]. Acting as an activator, PRAME interacts with transcription factor, NF‐Y, to promote the expression of genes involved in cell cycle progression and proliferation. PRAME can function as a repressor of RAR signalling, inhibiting retinoic acid mediated differentiation and apoptosis. Together, these mechanisms suggest that PRAME expression may promote tumour progression by enhancing proliferative signalling while also suppressing differentiation pathways.

Although these prognostication methods, and inclusion of mRNA PRAME expression has many benefits, they also have their limitations and no improvement in UM‐specific survival has been seen in the last 50 years [25]. Furthermore, these methods are not accessible to routine histopathology laboratories. It is costly, time‐consuming, and requires a specialised team, contributing to a two‐tiered model of care.

This study validated the use of prognostication markers such as PRAME and BAP1 IHC as a routine, rapid, cost‐effective alternative. Our results demonstrate that PRAME expression effectively identifies patients at risk for metastasis. By using IHC, we were able to achieve risk stratification that mirrors the prognostic value of GEP but within the capabilities of a routine diagnostic laboratory. Our work agrees with that of Han *et al*, whereby pairwise comparison of PRAME and BAP1 protein expression (PRAME±/BAP1±) by IHC was a better predictor of MFS and disease‐specific survival than TNM staging alone [17].

Han *et al* retrospectively examined BAP1 and PRAME IHC in 318 UM cases from a US cohort, stratifying tumours into four risk groups based on combined BAP1/PRAME status. Increasing risk group was associated with progressively higher rates of metastasis and disease‐specific death (DSS), with 5‐year MFS rates of 93%, 73%, 57%, and 28% across groups 1–4 respectively. Pairwise comparisons demonstrated that among BAP1 expressing tumours, PRAME positivity was significantly associated with worse MFS (HR 6.1, *p* = 0.001) and DSS (HR 8.3, *p* = 0.021), and among BAP1‐lost tumours, PRAME positivity was associated with shorter MFS (HR 2.4, *p* = 0.018). Critically, the combined BAP1/PRAME IHC profile outperformed pTNM staging alone as a predictor of metastatic outcome [17].

A key difference of our study from Han's group is the method of tissue sampling [17]. While Han *et al* utilised both TMAs and full sections without noting discrepancies, our study revealed a significant limitation of TMAs. We found that cases scored from a TMA were more likely to be negative (95%). We found that 21 tumours that stained negative on the TMA were found to be positive on whole sections (supplementary material, Figure S3). Our results recommend the use of whole‐section IHC over TMAs to account for tumour heterogeneity and focal expression hotspots that could potentially be contributing to the under stratification of high‐risk patients.

The identification of PRAME as an independent marker of increased metastatic risk in UM also has important therapeutic implications [25]. Demonstrating that PRAME expression identifies an intermediate risk subgroup highlights a cohort of patients who may be suitable for adjuvant therapies despite not showing traditional high‐risk features [16]. This is particularly relevant given the limited therapeutic options currently available for UM and the challenges associated with applying existing targeted approaches across all patients.

PRAME‐directed immunotherapies are beginning to show clinical relevance in UM. Immatics are currently evaluating a PRAME‐specific T‐cell receptor therapy, anzutresgene autoleucel (anzu‐cel), in the ongoing Phase 1b IMA203/IMA203CD8 trial, which includes patients with PRAME‐expressing solid tumours [26]. Preliminary results from the UM cohort, presented at the 2025 European Society for Medical Oncology and the Society for Melanoma Research meetings, demonstrated evidence of tumour shrinkage across treated patients (*n* = 16), with a reported median PFS of 8.5 months and manageable adverse effects [27]. Success was observed in patients who had previously failed treatment on tebentafusp, highlighting PRAME as a distinct target for overcoming acquired resistance in mUM.

Our results from 403 Irish/European UM patients confirm the results of the Han *et al* study of 318 UM cases from an American cohort, that UM can be effectively risk stratified using PRAME and BAP1 IHC stains. Our study validates the utility of performing PRAME IHC on UM full section tumours alongside established prognostic marker BAP1 to highlight patients who are at highest risk for metastatic spread. IHC is an accessible and affordable way for routine histology laboratories to assess patient risk promptly. Performing PRAME IHC also can identify which patients are eligible for emerging therapies without relying on longer, more expensive tests.

## Author contributions statement

SK, JC and NW conceptualised the study and oversaw execution. SO designed and executed the study, gathered clinical and histological information along with KH and GF, and analysed the data. SO and NW carried out statistical analysis. LI produced UM tumour TMAs. SK and SO scored all slides. NH and PM performed enucleations and provided tumour samples. SO wrote the paper and all authors had final approval of the submitted and published versions. Data is held by the Royal Victoria Eye and Ear Hospital and may be available from the corresponding author upon reasonable request and with permission from RVEEH.

## Supporting information


**Figure S1.** PRAME IHC, BAP1 IHC, and H&E staining
**Figure S2.** FISH images for various probes
**Figure S3.** Concordance of PRAME IHC expression: TMA and whole tumour sections
**Figure S4.** Survival analysis: stratified by PRAME TMA IHC+ chromosome 3 status
**Figure S5.** Survival analysis: stratified by nuclear BAP1 expression
**Table S1.** Uni/multivariate analysis on the NOPL cohort including PRAME
**Table S2.** Univariate analysis on the NOPL cohort for PRAME/BAP1 combination groups
