# Supplementary material for: PRAME predicting outcome in uveal melanoma
Source: J Pathol Clin Res. 2026 Oct 14;12(6):e70116. doi: 10.1002/2056-4538.70116 (PMC13662831; doi:10.1002/2056-4538.70116)
Supplement: Supplementary file 1 [file CJP2-12-e70116-s001.pdf]

PRAME predicting outcome in uveal melanoma

S Owens *et al.* *J Pathol Clin Res*. https://doi.org/10.1002/2056-4538.70116

Supplementary Figures S1–S5

Supplementary Tables S1–S2

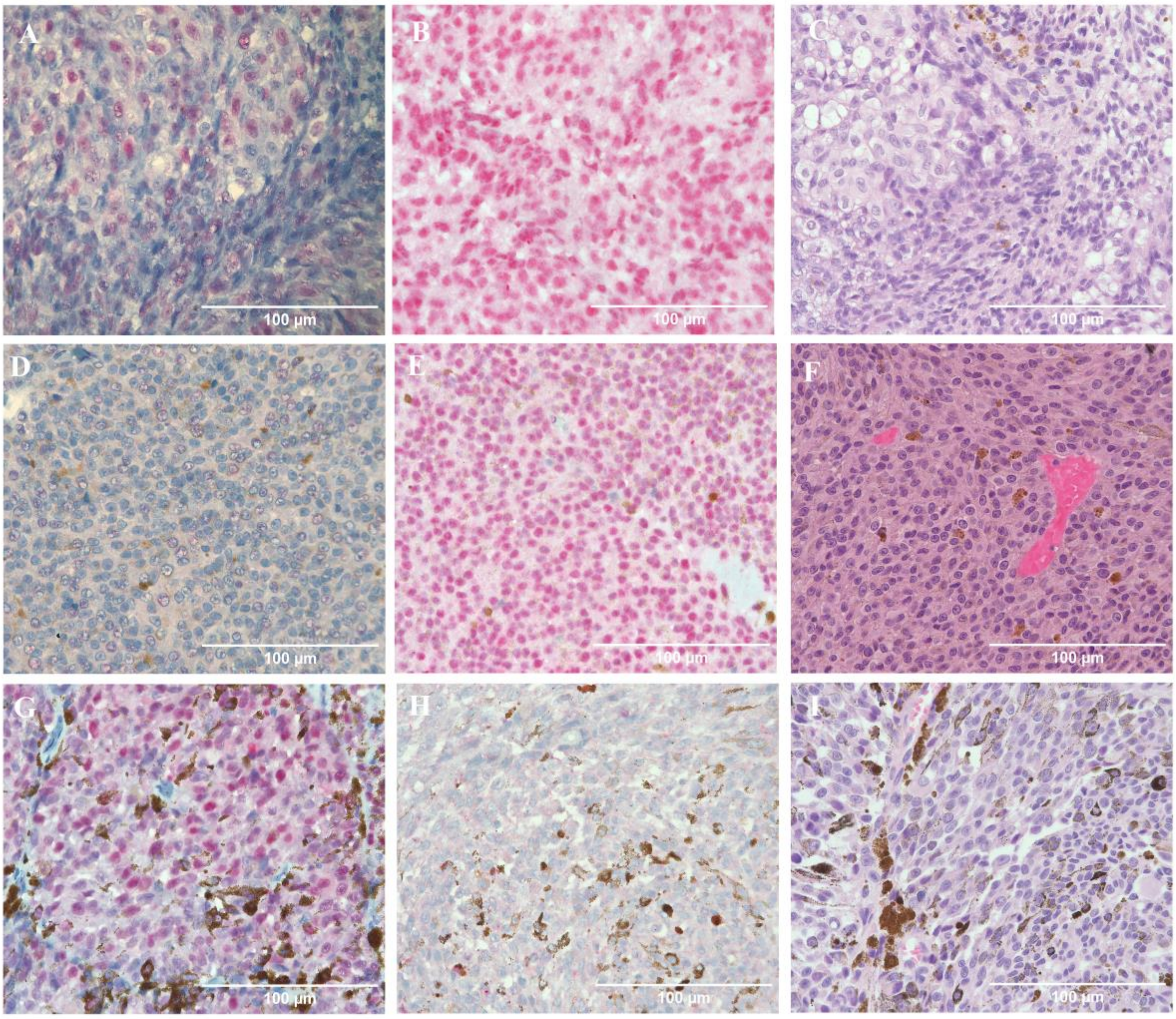

Figure S1. PRAME IHC (A, D, G), BAP1 IHC (B, E, H), and H&E staining (C, F, I) are shown for each case. In Case 1 (A–C), tumour cells show positive PRAME expression with retained BAP1 expression. In Case 2 (D–F), PRAME expression is lost with retained BAP1 expression. In Case 3 (G–I), PRAME is expressed and BAP1 expression is lost. Scale bars represent 100µm. All images were taken at 40x magnification.

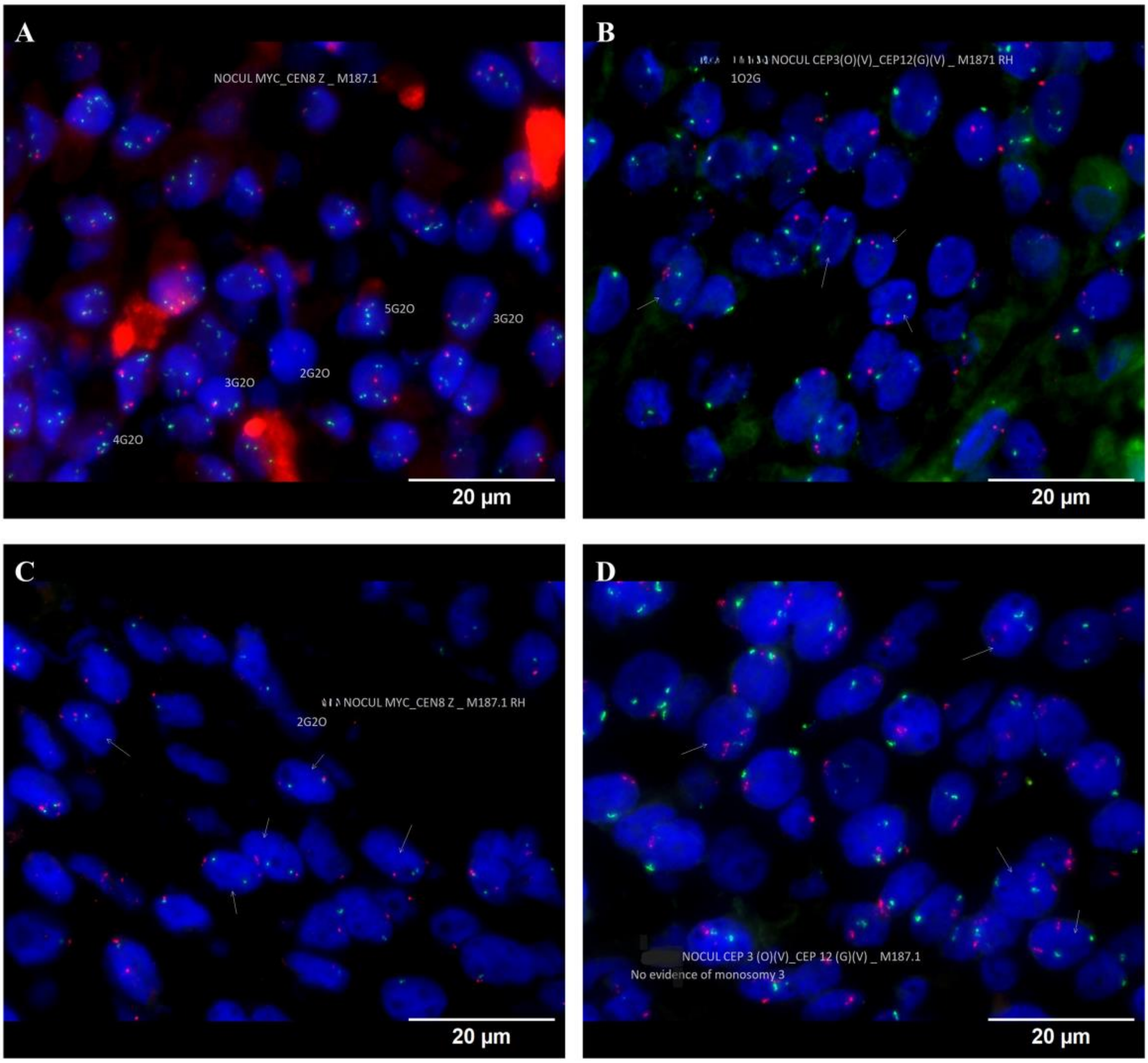

Figure S2. A) FISH using a locus-specific probe for MYC/8q24 (green) and a centromeric chromosome 8 probe (CEP8, orange, internal reference). Representative nuclei show three or more green signals relative to two orange reference signals (e.g. 3G2O, 4G2O, 5G2O), consistent with gain of the 8q24/MYC locus.
(B) Dual-colour FISH using centromeric probes for chromosome 3 (CEP3, orange) and chromosome 12 (CEP12, green, internal reference). Arrows indicate representative nuclei with a single CEP3 (orange) signal against two CEP12 (green) reference signals (1O2G), consistent with loss of one copy of chromosome 3 (monosomy 3).
(C) Same MYC/CEP8 probe combination showing representative disomic nuclei with two green and two orange signals (2G2O), consistent with no 8q gain.
(D) Same CEP3/CEP12 probe combination showing representative nuclei with two orange and two green signals, consistent with disomy 3 (no evidence of monosomy 3).
Nuclei are counterstained with DAPI (blue). Signals were enumerated manually in ≥100 non-overlapping tumour cell nuclei per case.

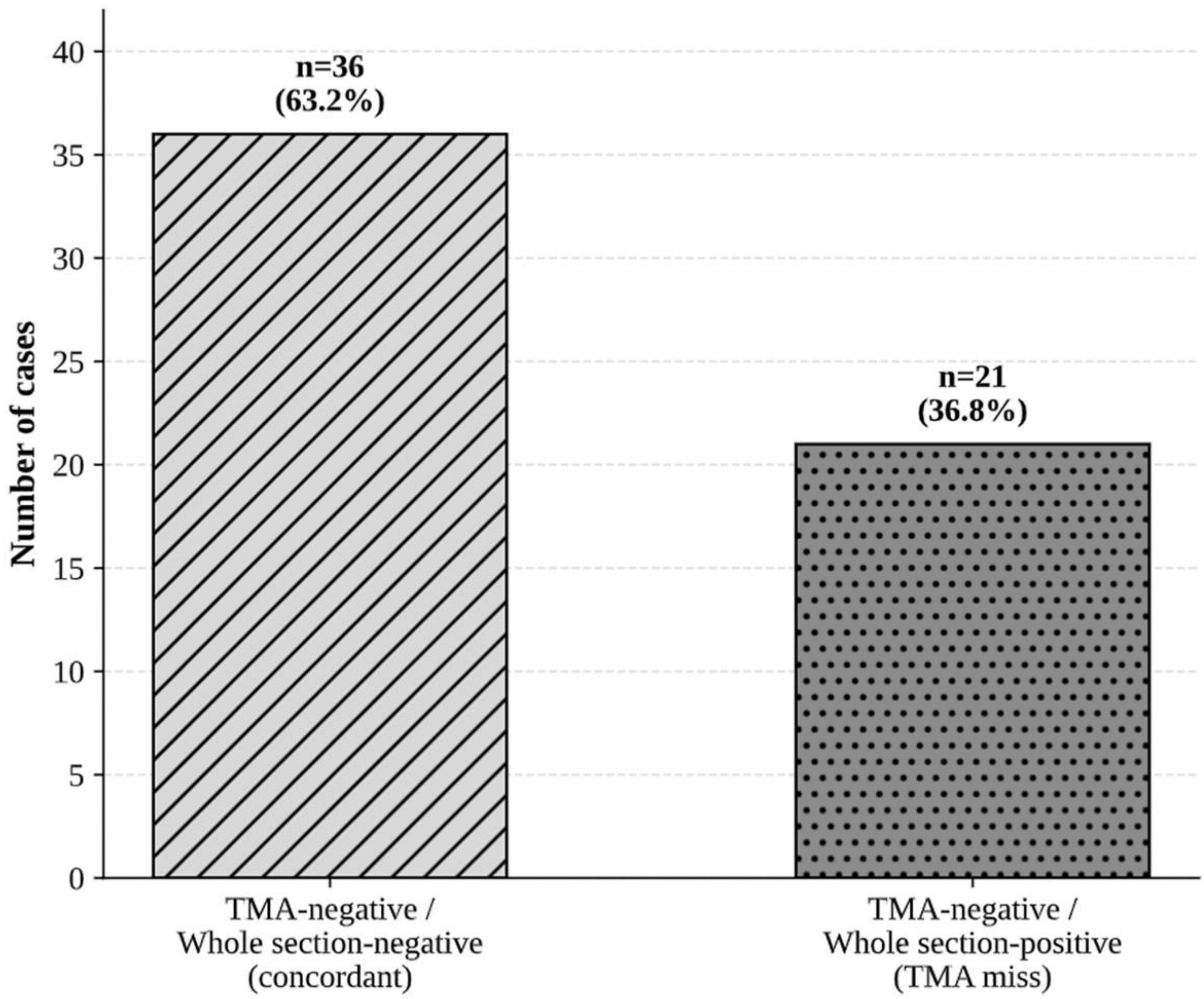

Figure S3. Concordance of PRAME IHC expression between tissue microarray (TMA) and whole tumour section in 57 paired cases. Bar chart showing the distribution of PRAME IHC scoring outcomes among the 57 cases with matched TMA and whole-section results. Of these, 36 (63.2%) were negative on both TMA and whole section (concordant negative), while 21 (36.8%) were negative on TMA but positive on whole-section IHC (TMA miss). No TMA-positive cases returned a discordant negative result on whole section in this paired subset.

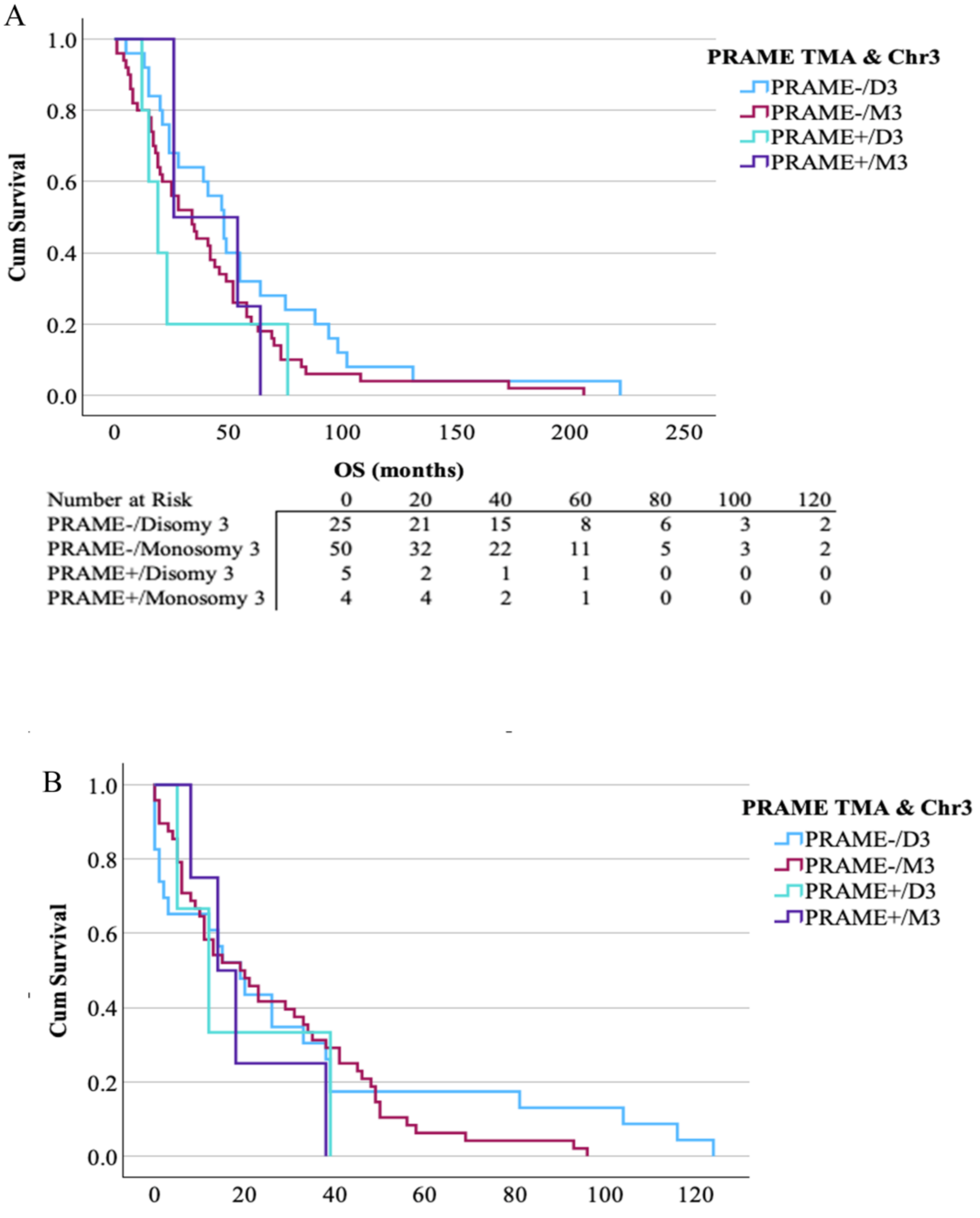

Number at Risk	0	20	40	60	80	100	120
PRAME-/Disomy 3	25	21	15	8	6	3	2
PRAME-/Monosomy 3	50	32	22	11	5	3	2
PRAME+/Disomy 3	5	2	1	1	0	0	0
PRAME+/Monosomy 3	4	4	2	1	0	0	0

Number at Risk	0	20	40	60	80	100	120
PRAME-/Disomy 3	19	11	4	4	4	2	1
PRAME-/Monosomy 3	46	24	14	3	2	0	0
PRAME+/Disomy 3	3	1	0	0	0	0	0
PRAME+/Monosomy 3	4	1	0	0	0	0	0

Figure S4. Kaplan-Meier A. overall survival (OS) and B. metastasis free survival (MFS) curves stratified by PRAME TMA IHC results combined with chromosome 3 status. PRAME-/disomy 3 (blue), PRAME-/monosomy 3 (red), PRAME+/disomy 3 (cyan) and PRAME+/monosomy 3 (purple). Time is portrayed on the x-axis in months. Log-rank tests were used to compare survival between the groups.

A

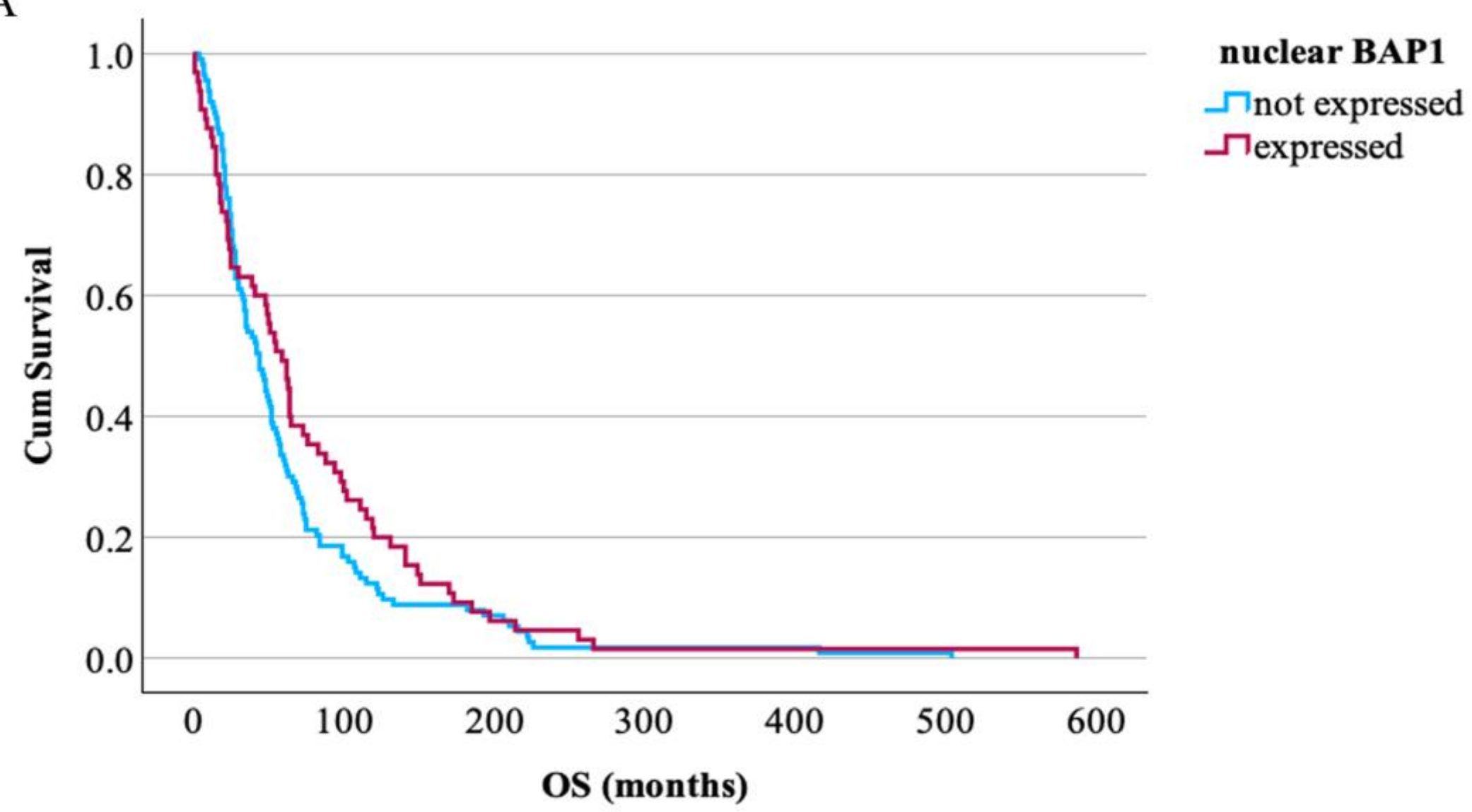

Number at Risk	0	100	200	300	400	500	600
nBAP1 not expressed	113	19	8	2	2	1	0
nBAP1 expressed	65	19	4	1	1	1	0

B

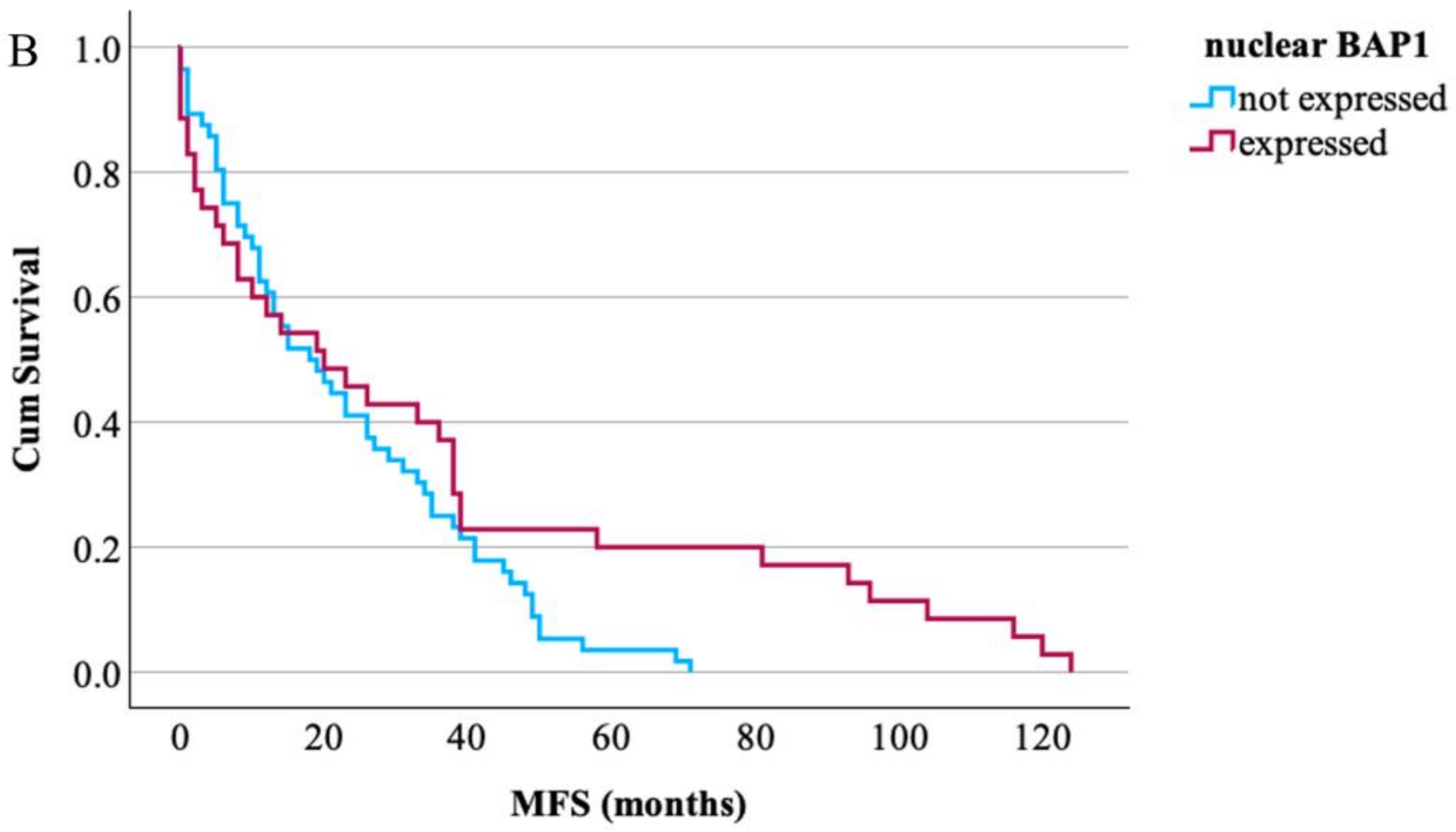

Number at Risk	0	20	40	60	80	100	120
nBAP1 not expressed	54	27	12	2	0	0	0
nBAP1 expressed	31	18	8	7	7	4	2

Figure S5. Kaplan-Meier A. overall survival (OS) and B. progression free survival (PFS) curves stratified by nuclear BAP1 positive expression (red) and negative expression (blue). Time is portrayed on the x-axis in months. Log-rank tests were used to compare survival between the two groups.

Table S1. Univariate analysis on the NOPL cohort incl. PRAME. Any significant variables were then included in the multivariate analysis. This was assessed for both OS and PFS. [NOPL: National Ophthalmic Pathology Laboratory; nBAP1: nuclear BAP1; TMA: tissue microarray]

	**Overall Survival (OS)**			
	**Univariate**		**Multivariate**	
**Characteristic**	**HR (95% CI)**	**p-value**	**HR (95% CI)**	**p-value**
**Age [> 62 years]**	1.535 (1.125-2.094)	**0.007**	0.332 (0.043-2.586)	0.292
**PRAME TMA**	1.901(1.049-3.443)	**0.034**	-	-
**PRAME full section**	2.369 (1.010-5.554)	**0.047**	1.809 (0.358-9.135)	0.473
**nBAP1 expression**	1.232 (0.904-1.678)	0.186		
**Ki67 expression**	1.627 (1.095-2.417)	**0.016**	2.680 (0.437-16.441)	0.287
**Monosomy Chr 3**	1.360 (0.863-2.142)	0.185		
**Chr8q gain**	1.489 (0.921-2.408)	0.104		
**AJCC pT1**	0.493 (0.308-0.790)	**0.003**	-	-
**AJCC pT2**	0.850 (0.560-1.292)	0.447		
**AJCC pT3**	1.272 (0.922-1.755)	0.143		
**AJCC pT4**	1.573 (1.071-2.311)	**0.021**	8.425 (1.204-58.976)	**0.032**
**Spindle cell type**	0.721 (0.521-0.999)	**0.049**	0.776 (0.146-4.121)	0.766
**Epithelioid cell type**	1.461 (1.047-2.038)	**0.026**	0.843 (0.247-2.871)	0.784
**Mixed cell type**	0.961 (0.718-1.288)	0.793		
	**Progression Free Survival (PFS)**			
	**Univariate**		**Multivariate**	
**Characteristic**	**HR (95% CI)**	**p-value**	**HR (95% CI)**	**p-value**
**Age [> 62 years]**	1.267 (0.829-1.936)	0.275		
**PRAME TMA**	1.412 (0.645-3.090)	0.388		
**PRAME full section**	8.447 (2.601-27.439)	**<0.001**	10.451 (2.385-45.798)	**0.002**
**nBAP1 expression**	1.496 (0.940-2.381)	0.089	3.877 (0.790-19.030)	0.095
**Ki67 expression**	1.075 (0.603-1.918)	0.806		
**Monosomy Chr 3**	1.165 (0.709-1.917)	0.547		
**Chr8q gain**	0.865 (0.508-1.473)	0.594		
**AJCC pT1**	1.394 (0.670-2.898)	0.374		
**AJCC pT2**	0.990 (0.547-1.791)	0.974		
**AJCC pT3**	0.642 (0.422-0.977)	**0.039**	0.72 (0.148-3.510)	0.684
**AJCC pT4**	1.724 (1.051-2.830)	**0.031**	0.624 (0.156-2.492)	0.505
**Spindle cell type**	0.728 (0.395-1.344)	0.31		
**Epithelioid cell type**	1.391 (0.899-2.152)	0.139		
**Mixed cell type**	0.955 (0.625-1.459)	0.831		

Table S2. Univariate analysis on the NOPL cohort for PRAME/BAP1 combination groups for OS. Note there was no deaths in the PRAME-/BAP1- group in the full section cohort.

PRAME TMA and BAP1 combination for OS		
	Sig.	Exp(B)
PRAME-/BAP1+ (reference)	.046	
PRAME-/BAP1-	.106	1.309
PRAME+/BAP1-	**.018**	**3.511**
PRAME+/BAP1+	.108	1.85

PRAME full section and BAP1 combination for OS		
	Sig.	Exp(B)
PRAME-/BAP1+ (reference)	.038	
PRAME+/BAP1-	**.011**	**4.447**
PRAME+/BAP1+	.491	1.499
